# Network Catastrophe: Self-Organized Patterns Reveal both the Instability and the Structure of Complex Networks

**DOI:** 10.1038/srep09450

**Published:** 2015-03-30

**Authors:** Hankyu Moon, Tsai-Ching Lu

**Affiliations:** 1 HRL Laboratories, LLC, 3011 Malibu Canyon Rd, Malibu, CA 90265-4797

## Abstract

Critical events in society or biological systems can be understood as large-scale self-emergent phenomena due to deteriorating stability. We often observe peculiar patterns preceding these events, posing a question of—how to interpret the self-organized patterns to know more about the imminent crisis. We start with a very general description — of interacting population giving rise to large-scale emergent behaviors that constitute critical events. Then we pose a key question: *is there a quantifiable relation between the network of interactions and the emergent patterns?* Our investigation leads to a fundamental understanding to: 1. Detect the system's transition based on the principal mode of the pattern dynamics; 2. Identify its evolving structure based on the observed patterns. The main finding of this study is that while the pattern is *distorted* by the network of interactions, its principal mode is invariant to the distortion even when the network constantly evolves. Our analysis on real-world markets show common self-organized behavior near the critical transitions, such as housing market collapse and stock market crashes, thus detection of critical events before they are in full effect is possible.

## Network self-organization as an indicator of critical transition

Self-organization has been understood as a non-equilibrium phenomenon of physical systems. (Here, we restrict our notion of self-organization to the ‘spontaneous order emergence’ due to a non-equilibrium phase transition.) Such phenomenon is characterized by the system's population transitioning from isolated behaviors to a persistent coordination due to the increasing instability near the transition. Ecosystems and human society also go through sudden regime shifts. T kinds of systematic shifts pose challenges to maintaining the stability of the nature or human society. Sudden disappearance of natural species could cause further disruption in the ecosystem. Unexpected opinion swings or market collapse poses hard challenges to the society. Efforts to detect and respond to such events before the onset of transitions are especially beneficial, because the measures to deal with any undesirable changes can be more effective before the system's full evolution toward a highly nonlinear regime^1^.

It has been speculated that catastrophic changes in nature are often preceded by peculiar signs^2^,^3^,^4^, such as regular-shaped patches of vegetation before desertification; recent studies^5^,^6^,^7^ investigated catastrophic population changes observed in ecosystems, and derived general quantitative indicators—*increased temporal correlation*, *skewness, and spatial correlations* of the population dynamics. Another study^8^ characterized a large-scale dynamical systems going through a bifurcation revealing self-organized spatial patterns as early signs. These approaches consider only the homogeneous lattice as the model of interactions. It would be a natural next step to investigate the self-organization of evolving complex networks—consisting of a large number of entities exchanging heterogeneous influences that can model a wide range of real world systems.

Despite its broad applicability, however, the model of ‘a heterogeneously networked dynamical system going through a phase transition’ has not been treated properly. The formidable issue of the *complexity of connectivity* has been mainly handled by statistical approaches—degree distributions, random network models^9^,^10^,^11^, etc. Order emergence due to spin dynamics^12^ or synchronization^13^,^14^ has been extensively studied without considering the evolving connectivity and phase transition at the same time. Only a recent investigation^15^ paid attention to the Turing patterns of complex networks governed by the specific case of activator-inhibitor dynamics, and another^16^ studied the interaction between network connectivity and dynamics.

The effective early warning signals introduced in Refs. 5,6,7 motivates us to develop a statistical measure that summarizes the collective dynamics of a networked population. In their model, each network node has been modeled as following a common dynamics, but the heterogeneous interactions in consideration may render highly complex patterns. The complexity naturally poses challenges in assessing where the system is and where it is heading. The key question is: *how does the network's structure affect its pattern of behavior at catastrophic regime shifts?* Our investigation on the complex network model leads to a very general indicator of a critical transition that is also not affected by its structure. While the heterogeneous network structure distorts the emergent pattern, the transition indicator (the pattern's principal mode) is invariant to the distortion even when the network constantly evolves with added/removed nodes and changing connectivity and weights. Moreover, the same analysis enables the estimation of the network's connectivity regardless of the particular system dynamics.

## Broken behavioral symmetry near phase transition causing self-organization

**Figure 1:**
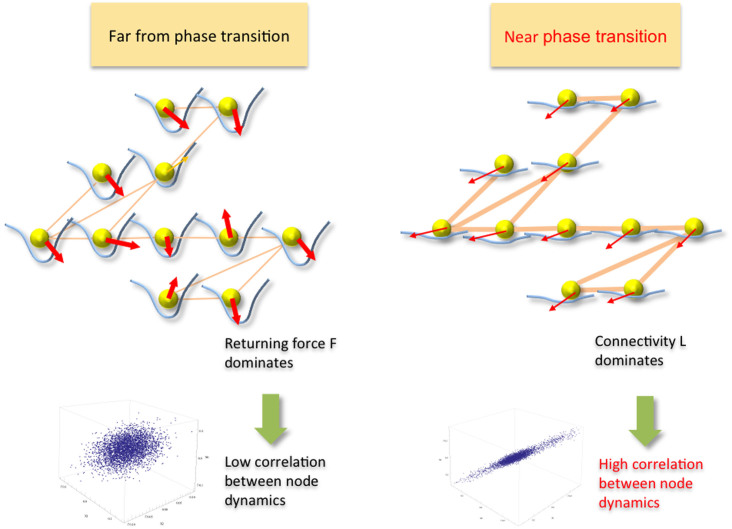
Self-organization due to instability before a critical transition. When the phase transition breaks the balance between the intrinsic dynamics and the peer influence, the effect of peer influence dominates and a self-organized population behavior emerges. Far from the transition, the dynamics 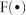 dominate over the relatively weaker interaction 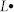
—exhibiting only individual motions. Near the transition, however, the returning force 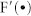 becomes much weaker than the persisting 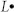 —the shifting balance from the dynamics to structure causes the nodes to become highly coordinated, resulting in global patterns.

There are many examples of complex networks showing self-organization. The formation and collapse of speculative market bubble have been largely regarded as the consequence of herd behavior. We postulate that in many cases the herd behavior emerges due to the *broken balance between autonomous behavior and peer influence*. Figure 1 illustrates the emergence of coordinated behavior. Every entity revolves around its own stable state while exchanging influence with peers or environments, maintaining the balance between these two effects. When a population of such entities goes through a system-wide change that weakens the intrinsic dynamics, the balance is broken and the effect of exchange propagates and dominates, which results in large-scale phenomena. It would then be possible to decode the emergent pattern to identify both the ongoing change and the *network of influence*.

Natural or social systems can be modeled as consisting of a population of entities (x_1_(t), x_2_(t), …, x_N_(t)) = X(t), exchanging material or informational influences through *networked interactions*. Each network node—an animal/plant or a person—constantly goes through changes in its state, such as metabolism, movement, or simply a daily cycle. It is natural to decompose the state changes x_i_ of the i-th node into two different components—one due to local *intrinsic dynamics* *f_i_*(x_i_) (*F*(*X*) = (*f*_1_(x_1_), *f*_2_(x_2_), …, *f_N_*(x_N_) for the whole network) and another due to a peer influence (The model only deals with undirected connectivity as in related studies^5^,^6^,^7^,^8^,^11^,^12^,^13^,^14^.) 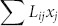, where L = {L*_ij_*} is the combinatorial *n* × *n* Laplacian matrix of the adjacency relations (degrees of interactions) *A*. L is computed by L = DegreeMatrix(A) − A. Each *f_i_*(x_i_) is a gradient to an equilibrium 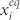
—an *attractor* (We only handle the mathematically more manageable case of a point attractor.) describing a stable behavior of x_i_. For instance, 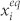 may represent a stable metabolic cycle or a person's well-established opinion about a certain topic. On the other hand, X is independently perturbed by a random Brownian motion σdW, which represents a fluctuation that constantly kicks each x_i_ slightly away from the 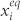. A *phase parameter* C augments the *f_i_*(.) to *f_i_*(.,*C*), capturing the network's evolution from a stable regime (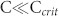) toward a different regime (C > C*_crit_*) via a transition point (C = C*_crit_*). The stability slowly decreases (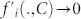) as C → C*_crit_*.

Some networks may evolve by an internal feedback, for example, the population adjusting their behavior based on the perceived system state. Then the phase parameter C is affected by the state X: 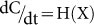, where H(X) represents the degree of the network's global order. For instance, media reports of rapidly increasing asset price can further drive the consumers to the market; H(X) may represent the price increase reflecting more consumers entering the market.

When the phase transition weakens the intrinsic dynamics, the effect of peer influence dominates and a self-organized population behavior emerges. Far from the transition, the dynamics *F*(*X*,*C*) dominate over the relatively weaker interaction LX—exhibiting isolated motions. Approaching the transition, the returning force *F*′(*X*, *C*) become weaker than the persisting LX. The shifting balance from the dynamics to structure causes the motions to become highly correlated, resulting in large-scale patterns. This is the well-known phenomenon of spontaneous symmetry breaking, which can also explain the self-organization of complex networks.

## Covariance spectrum revealing both instability and structure

Our main results are derived from this specific model (studied by many authors^5^,^6^,^7^,^8^,^12^), but are applicable to a broad range of complex networks where each node revolves around a stable state and exchange influences with other nodes—animal or human population is a prime example. The combination of the evolving dynamics, the peer influence, and the random perturbation leads to a network of stochastic differential equations: dX = (F(X,C) − LX)dt + σdW. The network's total deterministic dynamics around the equilibrium 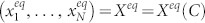 can be linearly approximated by the Jacobian of F(X, C) − LX at *X^eq^*:
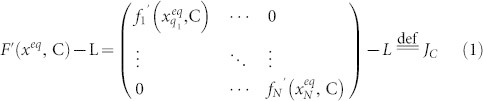


The degree of coordination is measured by the covariance matrix over some time window [t, t+Δt]: *Cov_c_*(t, X) = E_t,t+Δt_[X − Mean(X)][X − Mean(X)]^T^ for a fixed C. (In reality, C is assumed to change sufficiently slower than X.) A combination of the perturbation equation^17^ and its covariance equation^18^ derives [Supplementary Information 1] a closed-form solution of the *Cov_c_* in terms of the Jacobian and the Laplacian:
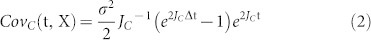


Let's assume that the returning forces become more homogeneous near the transition (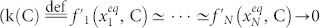, so that the covariance eigenvalues *μ*_1_,*μ*_2_,…,*μ_N_* can be expressed using the k(C) and the Laplacian eigenvalues *λ*_1_ = 0, *λ*_2_, …, *λ_N_*:
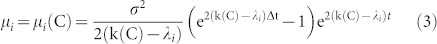


For a rigorous proof, please see [Supplementary Information 1].

The leading eigenvalue 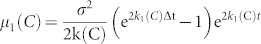 depends only on the *k*_1_(C) (but not on *λ*_2_, …, *λ_N_*)—implying that it can serve as a structure-invariant indicator of the transition. The invariance is a consequence of the peer interaction LX not contributing to the *synchronization manifold*^17^ *v_sync_* = (1,1,…1), but has nontrivial implications:

Property 1. The *degree of the global order μ*_1_ of the population indicates the network's instability due to a phase transition regardless of its connectivity.

Property 2. When the evolution is driven by an internal feedback 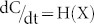, the resulting changes to the instability does not depend on the scale N or the connectivity L.

We conjecture that the first property would still hold without assuming the homogeneous returning forces 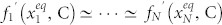, but we have been only able to derive the closed-form solution (3) and prove the invariance under this stronger assumption. Increasing order parameter is known to indicate instability due to a non-equilibrium phase transition, but our particular form of order parameter has the desirable property of structure-invariance. Though the ultimate goal is to forecast catastrophic events, instability seems more scientifically identifiable condition from which events may break out in combination with other factors.

So far we have assumed that the network Laplacian L is constant. The network itself may be allowed to change with the timescale much slower than the X (L is effectively fixed within the time window [t, t + Δt]). Still all of our results hold under the relaxed assumption. Property 1 leads to a corollary that *μ*_1_ is invariant to the evolution of the network—when it adds or removes nodes or edges (with changing weights) during the transition, *μ*_1_ will still provide a structure-invariant measure of the phase *C*. This property is especially powerful, because most real world networks go through constant changes in degrees of interactions. Property 2 holds because the ‘global order’ H(X) only contributes to the direction of *v_sync_*. It has an interesting interpretation in the context of the influence of newsfeeds—that the ‘destabilizing effect’ of global news (e.g., about the current state of the stock market) does not depend on the social network. (This property is verified in the Supplementary Information 2)

On the other hand, the rest of the covariance eigenvalues reveals the structure of the network. The function (3) is monotonically decreasing with the increasing Laplacian eigenvalues: 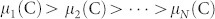. Therefore, the second largest mode *μ*_2_(C) represents the overall degree of the structure amplification. From equation (3), the instability will amplify the Laplacian spectrum *λ*_2_, …, *λ_N_* as k(C) → 0:

Property 3. The network reveals its underlying structure through the amplified Laplacian modes at the transition, facilitating the estimation of the network structure.

In fact, L can be recovered from the covariance matrices *Cov_C_*(t − Δt, X) and *Cov_C_*(t, X) computed over two successive time windows. Equation (3) derives:
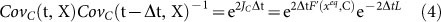


*F*′(*x^eq^*, C) is the only unknown factor. As can be seen from the expression (1), the heterogeneity of 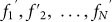 will distort the covariance eigenmodes. Near the transition, *F*′(*x^eq^*, C) → 0 so that the evolving connectivity *L* can be estimated more reliably independent of the particular *F*. That is, the distortion effect is zero at the transition—the dynamic network loses its individual property and its more genuine structure emerges.

## Instability and structure estimation of model networks at bifurcations

**Figure 2:**
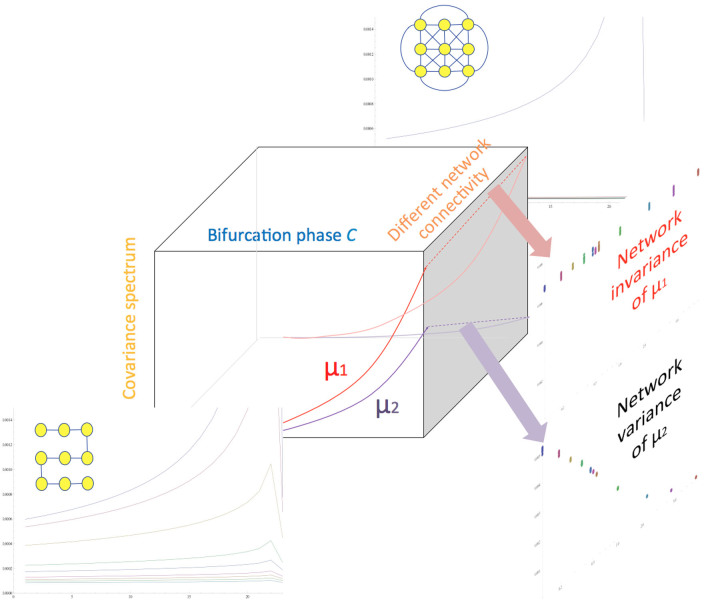
(Top and bottom) Difference in the growth of the covariance spectrum between a sparse (linear) network and a dense (fully connected) network. The top plot shows the growth of the covariance spectrum from a linearly connected network as it evolves toward a fold bifurcation. The top plot shows the spectrum growth for a fully connected network as it goes through the same evolution. Both *μ*_1_s (top curves) reveal identical growths (verifying the invariance), but the rest of the spectrum (curves beneath each top curve) show vastly different growth profile. Note that higher order spectrum for the fully connected network is collapsed into almost flat curves. (Right) Invariance and variance over 10 different network topologies. The upper plot shows uniform growths of *μ*_1_ computed over 10 networks having varied connectivity, verifying the invariance. The lower plot shows the varied growths of *μ*_2_ computed for the same set of networks.

The derived relation between the network connectivity and the covariance matrix in equation (2) holds regardless of the F(.,*C*)), as long as it goes through a local bifurcation—a general mathematical model of phase transition. The fold bifurcation 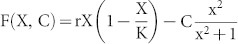 models^5^,^6^,^7^ the catastrophic changes of logistically changing population, with the shifting grazing rate C, the growth rate r, and the carrying capacity K. The pitchfork bifurcation F(X, C) = CX − X^3^ models the transition from a single-well potential to a double-well potential—an emergence of alternative regimes. There are other forms of bifurcation, but the diversity is reduced near the C = C*_crit_* to exhibit the same qualitative behavior. Figure 2 shows the growth of covariance spectra for a linearly connected 9-node network (bottom) and a fully connected 9-node network (top), all going through fold bifurcations. The *μ*_1_(*C*) evolves identically for both networks, but the rest of the spectrum follows different curves as expected by the equation (3). The growth of covariance spectra from ten different networks having varied connectivity confirms the invariance property of the *μ*_1_(*C*) and the variance property of the *μ*_2_(*C*).

**Figure 3:**
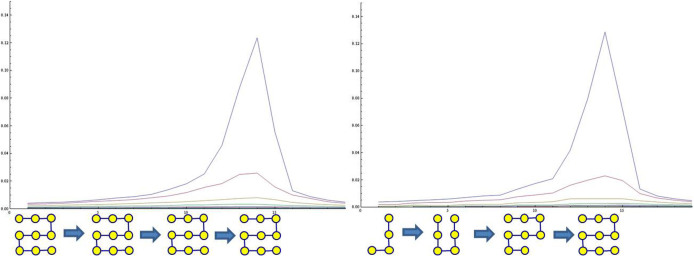
Invariance of *μ*_1_ for a growing network. The horizontal and vertical axes represent the time evolution of the networks toward a pitchfork bifurcation and the changes in spectrum, respectively. The invariance of the leading eigenvalue *μ***_1_** as an indicator of phase transition holds while network nodes and edges are being added or removed. (Left) The plot is the spectrum changes of a 9-node linear network as it goes through a pitchfork bifurcation, provided as a reference. The linear structure is fixed during the network's evolution. The spectrum is ordered 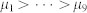 from the top curve. (Right) The plot is the spectrum changes of a growing network, starting from a 4-node linear network to a 9-node linear network. At each growth stage, the covariance matrix is constructed from the augmented nodes; the covariance matrix starts from a 4 × 4 matrix and grows to a 9 × 9 matrix. The leading eigenvalues from both plots show almost identical curves, confirming the invariance of *μ***_1_**.

The property 1 supports the invariance of the leading eigenvalue while network nodes and edges are being added or removed, and the Figure 3 verifies it. The left plot is provided as a reference: the spectrum changes of a 9-node linear network when its structure is fixed while going through a pitchfork bifurcation. The right plot shows the spectrum changes of a growing network, when it grows from a 4-node linear network to a 9-node linear network by adding one node and one edge at a time. Both leading eigenvalues *μ*_1_ show identical growths.

**Figure 4:**
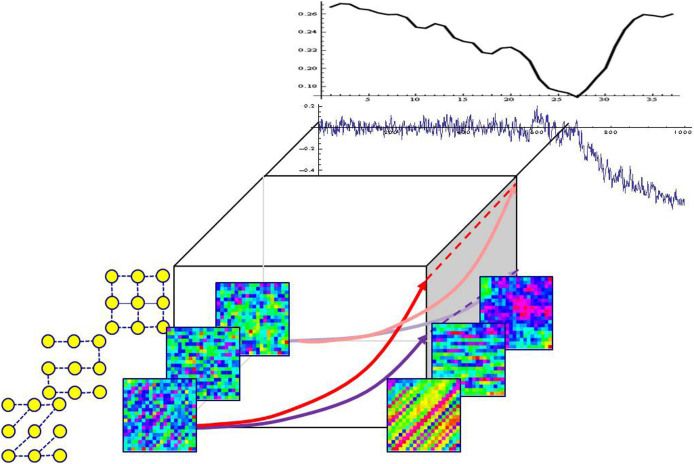
Structure revealing pattern formation property. (Bottom) The horizontal axis represents the time evolution of the network going through a pitchfork bifurcation. Three 20 × 20 networks having distinct geometric connections—grid, linear, and slanted—goes through the bifurcation. When each network is far from the bifurcation, the state of the 20 × 20 = 400 node network represented as pixel values show only slight differences. However, when each network approaches the bifurcation point, the pattern clearly reveals the underlying geometry. (Top 2 plots) Structure estimation at phase transition. 9-node linear network dynamics was simulated to go through a pitchfork bifurcation (lower plot), and the equation (4) estimates the Laplacian matrix from the state covariance matrices. The difference in terms of Frobenius norm between the estimated Laplacian matrix and the true Laplacian matrix is plotted (upper plot). The difference achieves a minimum at the bifurcation point, verifying the optimal structure recovery property at the phase transition.

The structure revealing property at phase transition is shown in Figure 4. In the bottom inset figures, three distinct geometric network structures in two-dimensional 20 × 20 grids are compared to illustrate different pattern emergence. When each network is far from the pitchfork bifurcation, its 20 × 20 = 400 nodes (represented as pixel values) show only slight difference. However, when they approach the bifurcation point, the pattern clearly reveals the individual differences in geometry. (The same property for large-scale random networks is verified in the Supplementary Information 3.) In the top panel, 9-node linear network dynamics was simulated to go through a pitchfork bifurcation. Equation (4) is used to estimate the Laplacian matrix from the state covariance matrices. The Frobenius norm between the estimated Laplacian matrix and the true Laplacian matrix is plotted (upper plot); the difference achieves a minimum at the bifurcation point, verifying the optimal structure recovery property at the bifurcation. The next two sections reveal that real-world housing and financial markets show common self-organized behaviors near phase transitions, and are measurable using the method derived from the same generic network model.

## Consumer herd behavior in housing market

Human society is being modeled and studied as a complex system to identify trends or crises, further encouraged by the recent surge of publicly available data. One study^19^ focused on the behavior of mimicry in trading decisions based on price fluctuations, while another^20^ utilized information-gathering behavior of traders by monitoring Web search volumes. These behaviors fit the profile of our behavior model; however, they utilize conventional scalar quantity (price index, search volume, etc.) as the measurement of the population behavior. Our analysis interprets multiple measurements based on an underlying dynamic model to detect trends or crises.

**Figure 5:**
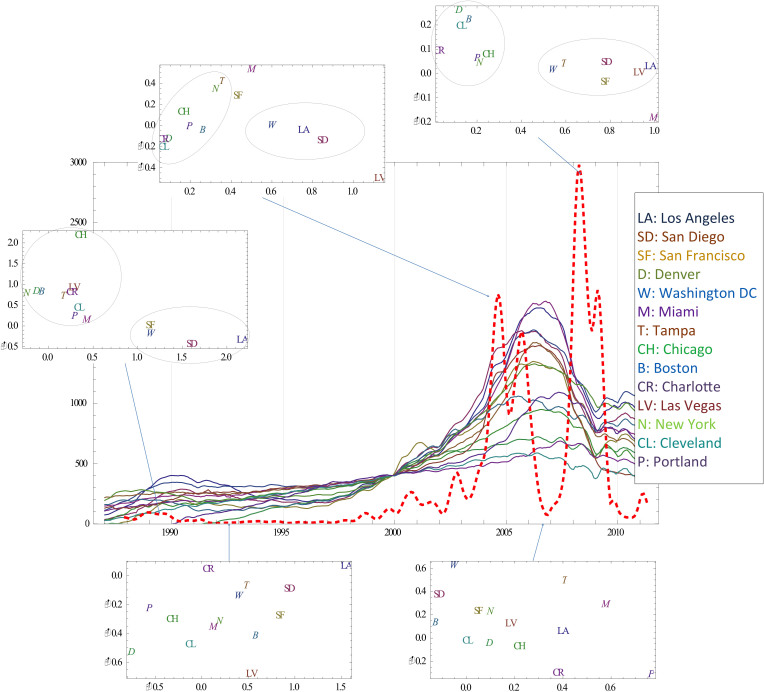
Housing market instability. (Central plot) The changes in housing price and the covariance spectrum as a leading indicator of market instability. The horizontal axis represents the time evolution of the system from 1987 to 2011. The red dotted curve is the instability measure *μ*_1_ of the housing market, computed from the price changes over 14 cities (colored plots). The rapid price increase and collapse coincide with a very high degree of instability, especially during the latest housing boom (2003–2006) and bust (2007–2009). (Insets) Structure recovery at phase transition. Each inset shows the projections of the 14 cities into the two-dimensional space during a different period, where each city's coordinate is the corresponding first and the second eigenvector components. The distribution of the cities during the market instability (three top panels) show expected clusters of markets. Most distinctively, Los Angeles, San Diego, and Washington DC stay close during three different periods (1988 bubble, 2004 bubble, and 2008 collapse). We suspect that San Francisco behaved differently during the 2004 peak, because of the lasting impact of the earlier dot-com collapse. The rest of the cities form another cluster. Three new ‘bubble cities’—Las Vegas, Miami, and Tampa—disrupt the cluster structure during the recent housing bubble. We are not concerned about explicit causal influences between market moves; the proximity may indicate true correlations or suggest that they merely follow similar ‘bubble market’ behavior. In contrast, the periods when the market stabilized (1992) or the market tried to find a direction (2006), the projections (bottom panels) do not show any meaningful clusters.

Housing bubble is often attributed to consumers' herd behavior. Their buying or selling decisions are made based on both personal circumstances and peer effects. We postulate that external influence (e.g., low mortgage rates) destabilize individuals to base their purchase decisions less on personal circumstances but more on the others' decisions. In Figure 5, the changes in housing market indices (Case-Shiller index^21^) since 1987 of 14 major US cities have been utilized as reflecting the consumer behavior^22^. Case-Schiller Index covers 20 cities, but the study excluded 6 cities due to incomplete data. The curve in red dashes depicts the *μ*_1_, computed from the 14 indices (colored plots). The coordinated price increase and collapse coincide with a very high degree of instability measured by the *μ*_1_, especially during the latest housing boom (2003–2006) and bust (2007–2009). Though each index is a somewhat noisy measurement of the local consumer behavior, the *μ*_1_ does show very high degrees of market correlation. On the other hand, we expect that an unstable market would reveal the underlying network structure more reliably than a stable and de-correlated market would. Each inset shows the projections of the 14 cities into the two-dimensional space during a different period, where each city's coordinate is the corresponding first and the second eigenvector components. The distribution of the cities during the market instability (three top panels) indeed shows expected clusters of markets. In contrast, the periods when the market stabilized (1992) or the market tried to find a direction (2006), the projections (two bottom panels) do not show any meaningful clusters. Most interestingly, Washington DC stayed close to Los Angeles and San Diego during three different periods (1988 bubble, 2004 bubble, and 2008 collapse). We suspect that San Francisco behaved differently during the 2004 peak, because of the lasting impact of the earlier dot-com collapse. (San Francisco, Los Angeles, and San Diego belong to the same state of California.) The rest of the cities form another cluster. New ‘bubble cities’—Las Vegas, Miami, and Tampa—disrupt the cluster structure during the recent housing bubble. These three cities went through the most severe bubble cycles during the 2003–2008 housing bubble and crash. We are not concerned about explicit causal influences between the markets; the proximity may indicate true correlations or just similar local market characteristics.

## Self-organized trader behavior causing large-scale stock price moves

Financial markets are also driven by collective behaviors of traders. Each trader makes decisions after absorbing information—not only the information about external factors—economic picture, political change, world events, etc.—but also the information about the state of the market, which is the sum of all traders' decisions. Any information of predictive values quickly feeds back to the system, affects trading, and is ultimately reflected to the price (EMH^23^). If the market is truly efficient, such instant information feedback strips the price dynamics of any trends, allowing only random fluctuations; random walk is a popular model of financial market dynamics. If stock markets do follow pure random walks, then prediction is essentially not possible. However, there have been counter arguments and efforts to compromise the EMH with behavioral economics^24^. Some of these investigations^25^ observe that there are transient temporal correlations during financial bubbles or crashes, whereas others^26^,^27^ investigate increasing cross-correlation within share movements. More recent work^20^,^22^,^28^,^29^ employs physics based models of self-organized emergent trader behavior to explain extreme market moves.

This type of non-stationary dynamic models could compromise the random behavior (due to the efficiency) and ordered behavior of market dynamics. Our analysis also supports the same conclusion that the market shows increased correlation among share prices before and during extreme market events. Our dynamic model provides computational tools to capture such emergent market behaviors. While increased market synchronization is commonly observed before and during market crashes^26^,^27^, our work firmly establishes the first covariance eigenvalue *μ*_1_ of share prices as a mathematically grounded measure of market instability. Another study^30^ found empirical evidence that co-movements of share prices over long-term periods (over a month) precede market crashes. For the empirical evaluations shown below, a combination of two *μ*_1_′s from both short-term and long-term market correlations. The [Supplementary Information 4] provides details of the evaluation methodology.

**Figure 6:**
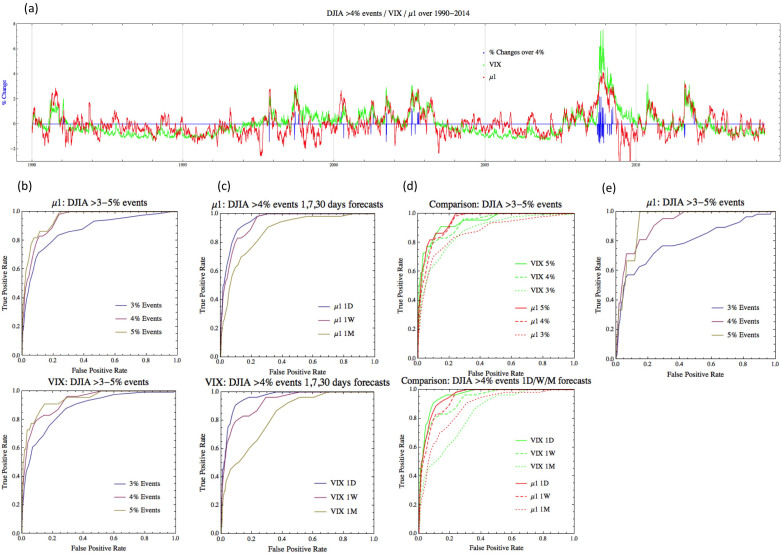
The leading covariance eigenvalue as an indicator of instability of stock market. (a) The plot of percentage changes of DJIA between 1990 and 2014 that are greater than 4% (blue spikes) and the computed leading covariance eigenvalue *μ***_1_** (red curve). The VIX (Chicago Board Options Exchange Market Volatility Index) is plotted (in green) as a comparison. Most of the large spikes during the 24-year period occurred while the *μ***_1_** is high. The *μ***_1_** and VIX follows each other closely, especially during high-volatility periods. (b: top) ROC (Receiver Operating Characteristics) curve when *μ***_1_** was used as a simple decision function for predicting market events in a week (five trading days). The horizontal axis represents false positive rate (FPR) and the vertical axis represents the true positive rate (TPR). The plot shows that *μ***_1_** detects more than 80% of the market events while tolerating less than 20% false positives. Different event definitions (3%, 4%, and 5%) are compared and they indicate that 5% events are more predictable than 3% events. (c: top) Comparison between different prediction horizons: 1-day, 1-week, and 1-month. The performances degrade for longer prediction horizons, but very modestly. (b,c: bottom) The ROC curves for VIX are plotted below the corresponding *μ***_1_** ROCs. For better comparisons they are shown in the same plot in (d): (d: top) shows that the two methods are comparable for 1-week prediction performances over different event classes, but *μ***_1_** still does slightly better for the 4% and 5% event classes. (d: bottom) reveals that *μ***_1_** performs noticeably better for 1-month prediction, indicating that VIX may be more reactive than predictive. (e) ROC for the period of 1990–2007 to verify if the method works for more ‘modest crashes’. It shows detection performance for the 4% and 5% events not much different from the whole period including the 2008 crash (b: top), verifying the generality of the method.

Figure 6 summarizes the result where the share price correlation of DJIA (Dow Jones Industrial Average) measured by *μ*_1_ is used as an indicator of significant ‘market events’, defined as rare, large-scale daily changes of DJIA. The daily returns of the closing prices of each share (such as IBM, GE, etc. that belongs to DJIA) are the input time series to the computation. Figure 6 (a) compares the timeline of Dow Jones market events (daily change over 4%, marked with blue spikes) from 1990 to 2014 and the computed *μ*_1_ (red plot). Most of the large spikes during the 27-year period occurred while the *μ*_1_ is high.

We then used the *μ*_1_ as a simple decision function for predicting market events: after each trading day, if the computed *μ*_1_ crosses a certain threshold, we predict that a DJIA change (drop or jump) larger than 4% will occur in a fixed prediction horizon (one day, one week, or a month). Figure 6 (b: top) show the ROC (Receiver Operating Characteristics) curve for the one-week (five trading days) prediction performance. The horizontal axis represents the false positive rate (FPR) and the vertical axis represents the true positive rate (TPR). The plot shows that *μ*_1_ detects more than 80% of the market events while tolerating less than 20% false positives. It compares different event definitions, in terms of magnitude of index changes: 3%, 4%, and 5% jump/drop events. It indicates that extreme events (5% events) are more predictable than more common events (3% events). The Figure 6 (c: top) compares different prediction horizons: one-day, one-week, and one-month (22 trading days as an approximation) predictions. While the accuracy degrades for longer prediction horizons, one-month prediction still shows much higher detection rates than random guesses (the curve will be reduced to the diagonal line of TPR = FPR).

One can observe that the historic crash of 2008 involved a series of large-scale events, as also shown in the Figure 6 (a), where the market panic due to the financial sector trouble caused mass selling across the board, further causing multiple large scale stock moves. The first a few days of significant drops surprised investors, but following market instability may have been well expected. Therefore, we have removed the period of extreme market moves and computed the ROC (Figure 6 (e)) for the period of 1990–2007 to verify if the method works for more ‘modest crashes’. The ROC curve for the period shows detection performance for the 4% and 5% events not much different from the whole period including the 1987 and 2008 crashes, verifying the generality of the method.

We also made a comparison to the VIX (Chicago Board Options Exchange Market Volatility Index^31^), the most popular and successful market volatility measure. The VIX represents the market's expectation of stock market volatility over the next 30-day period. The green curve of the Figure 6 (a) is the timeline of VIX, scaled for a better comparison to the *μ*_1_. Overall these two independently developed measures closely follow each other, especially during highly volatile periods. Figure 6 (b,c: bottom) shows the ROC curves for VIX are plotted below the corresponding *μ*_1_ ROCs. For better comparisons they are shown in the same plots in (d). (d: top) shows that the two methods are comparable for 1-week prediction performances over different event classes, but *μ*_1_ still does slightly better for the 4% and 5% event classes. (d: bottom) reveals that *μ*_1_ performs noticeably better for 1-month prediction. Given that VIX achieves slightly better 1-day performance, we could conjecture that VIX is more reactive than predictive, compared to our method.

**Figure 7:**
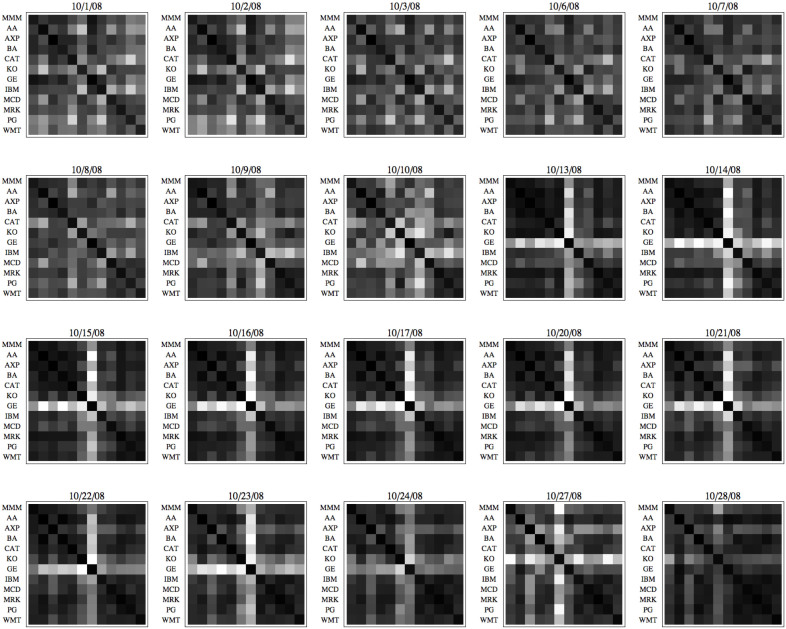
Correlation pattern dynamics as indicators of large-scale market crash. Stock market shows peculiar patterns before large-scale crashes, as demonstrates for the 2008 crash. The market crash happened on October 15, 2008, leading to the DJIA losing 7.9%. The square blocks visualize the sequence of covariance matrices from twelve share prices (subset of the DJIA constituents) before (10/1/2008–10/14/2008) and after (10/15/2008–10/28/2008) the crash; darker pixels represent higher pair-wise correlations between the share price movements. The diverse correlations as shown in the top row represent typical market behavior weeks before the event. However, staring 10/13 (the second row) not only the overall correlation increased but the pattern persisted through the following week (the third row) during and after the event (10/15). More examples are provided in Supplementary Information 4.

As we have discussed the mathematical model of pattern formation equation (4) and provided evidence in housing market example, stock market also does show peculiar patterns before large-scale crashes—not only the increase of overall correlation as captured by *μ*_1_. Figure 7 demonstrates the correlation pattern as a potential crash indicator during the 2008 crash. The biggest single-day crash (among other crashes during the period) happened on October 15, 2008, leading to the DJIA losing 7.9%. The figure shows an interesting market behavior prior to the crash and during the crash. The square blocks visualize the sequence of covariance matrices from twelve share prices (subset of the DJIA constituents) before (10/1/2008–10/14/2008) and during/after (10/15/2008–10/28/2008) the crash; darker pixels represent higher pair-wise correlations between the share price movements. The diverse and changing correlations as shown in the top row represent typical market behavior weeks before the event. However, staring 10/13 (the second row) not only the overall correlation increased (the *μ*_1_ plot in Figure 6 captures it) but the pattern persisted through the following week (the third row) until the crash date (10/15). We do observe similar behavior of persistent pattern dynamics during the 1987 Black Monday crash, and other significant market events (Supplementary Information 5). The market behavior after the 9/11/2011 terrorist attack is also provided as an interesting example, where the purely exogenous crash, while large in magnitude, does not show the signature self-organized patterns. The observed behavior strongly agrees with our theory finding summarized in equation (4)—that a phase transition does amplify the underlying structure so that the emergent pattern will be more explicit and persistent. The principal components from market correlation have been investigated previously for identifying business sectors^32^ and discovering evolution of market influences^26^. We have investigated the same phenomena at finer time scales to reveal that the emergent patterns not only become prominent during crises, but also predictive and persistent.

## Discussion

We have developed a mathematical model of a complex network going through a phase transition to derive a predictive measure of such transition based on the observed time series from the network. Under this model, the first eigenvalue of the covariance matrices (computed from the time series) is mathematically proven as a network-invariant indicator of instability. The same model also suggests another indicator—persistent patterns in time series correlation. Experiments on historical housing and stock market data confirm that the eigenvalue significantly increases before large-scale market events, and persistent pattern dynamics are also observed as preceding signs. Many literatures have discussed collective behaviors as potential signs of catastrophic events, but only recent work^5^,^6^,^7^ developed quantitative indicator to be applicable to real-world data. We also believe that increased correlation and pattern dynamics are general behaviors of complex networks; therefore, the proposed computational tool should find broader applications. Our framework is a very early effort to establish a mathematically grounded indicator for analyzing time series data from networked entities, and to verify applicability to real-word data. As our initial success indicates, the computational method in the proposed form should be applicable to data from different domains. On the other hand, the model is general enough to allow domain-specific dynamic models to be incorporated to develop more specialized indicators of instability.

Further investigation should also focus on deriving a quantitative measure of the pattern dynamics. In general, spectral analysis of fluctuations separates the dynamics of the magnitude (captured by eigenvalues) from the modes (captured by eigenvectors). Therefore, there is a potential to have an improved predictor by combining these two separate quantities. Additionally, further investigations to the whole spectrum of the covariance (not just the first eigenvalue) may provide insights into the scaling behavior of complex networks—how correlation strengths at different scales that is precisely measured by the spectrum are related to instability of complex networks. We also hope to find suitable spatiotemporal data collected from natural systems to test our method, as in the related work^5^,^6^,^7^ applied to ecological data.

## Supplementary Material

Supplementary Information Supplementary information
