# Supplementary material for: Network Catastrophe: Self-Organized Patterns Reveal both the Instability and the Structure of Complex Networks
Source: Sci Rep. 2015 Mar 30;5:9450. doi: 10.1038/srep09450 (PMC4378512; doi:10.1038/srep09450)
Supplement: Supplementary Information [file srep09450-s1.pdf]

# Network Catastrophe: Self-Organized Patterns Reveal both the Instability and the Structure of Complex Networks

Hankyu Moon and Tsai-Ching Lu
HRL Laboratories, LLC
3011 Malibu Canyon Rd
Malibu, CA 90265-4797
{hmoon, tlu}@hrl.com

## 1. The derivation of the covariance matrix and eigenvalues

The stochastic differential equation that represent the complex network dynamics is given by $\mathrm{dX} = (\mathrm{F(X, C)} - \mathrm{LX})\mathrm{dt} + \sigma \mathrm{dW}$. The changes of the node states are represented by $\mathrm{X} = \mathrm{X(t)} = (\mathrm{x_1(t), x_2(t), \dots, x_N(t)})$ whose intrinsic dynamics are governed by $F(X, C) = (f_1(\mathrm{x_1}, C), f_2(\mathrm{x_2}, C), \dots, f_N(\mathrm{x_N}, C)$ slowly going through phase transition as $\mathrm{C} \to \mathrm{C_{tran}}$. Linear peer influence $\sum \mathrm{L}_{ij} x_j$, where $\mathrm{L} = \{\mathrm{L}_{ij}\}$ is the Laplacian matrix of adjacency relations $A$: $\mathrm{L} = \mathrm{Degree(A)} - \mathrm{A}$, also affects the node dynamics as well as the random perturbation $\sigma\mathrm{dW}$.

The system's total deterministic dynamics around the equilibrium $X^{eq} = (x_1^{eq}, \dots, x_N^{eq})$ can be linearly approximated by the Jacobian of $\mathrm{F(X, C)} - \mathrm{LX}$ at $X^{eq}$:

$$F'(x^{eq}, \mathrm{C}) - \mathrm{L} = \begin{pmatrix} f_1'(x_1^{eq}, \mathrm{C}) & \cdots & 0 \\ \vdots & \ddots & \vdots \\ 0 & \cdots & f_N'(x_N^{eq}, \mathrm{C}) \end{pmatrix} - \mathrm{L} \stackrel{\mathrm{def}}{=} J_C \quad \text{(S-1)}$$

To compute the covariance matrix over some time window $[\mathrm{t}, \mathrm{t} + \Delta\mathrm{t}]$: $P(\mathrm{t}, \mathrm{t} + \Delta\mathrm{t}) = \mathrm{E}_{\mathrm{t,t+\Delta t}}[\mathrm{X} - \mathrm{Mean(X)}][\mathrm{X} - \mathrm{Mean(X)}]^{\mathrm{T}}$ for a fixed C, the behavior of $\mathrm{F(X, C)}$ around the $X^{eq}$ can be well approximated by a linearization at the equilibrium following a standard perturbation method [13]:

$$\mathrm{d}\xi = J_C \xi \, \mathrm{dt} + \sigma \mathrm{dW} \quad \text{(S-2)}$$

By [14] the covariance matrix $P(t) = \int_0^t [\xi - \mathrm{Mean}(\xi)][\xi - \mathrm{Mean}(\xi)]^T ds$ satisfies a deterministic differential equation $\mathrm{dP} = 2J_C \mathrm{P} \, \mathrm{dt} + \sigma^2 I$, whose solution is:

$$P(\mathrm{t}) = \frac{\sigma^2}{2} {J_C}^{-1} (\mathrm{e}^{2J_C \mathrm{t}} - 1) \quad \text{(S-3)}$$

Now, the covariance matrix $P(\mathrm{t}, \mathrm{t} + \Delta\mathrm{t})$ over the sliding time window is calculated by

$$P(\mathrm{t}, \mathrm{t} + \Delta\mathrm{t}) = \mathrm{P(t + \Delta t)} - \mathrm{P(t)} = \frac{\sigma^2}{2} {J_C}^{-1} (\mathrm{e}^{2J_C \Delta\mathrm{t}} - 1) \mathrm{e}^{2J_C \mathrm{t}} \quad \text{(S-4)}$$

The assumption $f_i'(x_1^{eq}, \mathrm{C}) \simeq \cdots \simeq f_N'(x_N^{eq}, \mathrm{C}) \stackrel{\mathrm{def}}{=} \mathrm{k(C)}$ enables the computation of covariance eigenvalues from (S-4), because $J_C = F'(x^{eq}, \mathrm{C}) - L = \mathrm{k(C)} \cdot \mathrm{I} - \mathrm{L}$ and the two terms can share the common eigenvectors. Because all the matrices in (S-3) commute and share the common eigenvectors, the eigenvalues $\mu_1, \mu_2, \dots, \mu_N$ are expressed in terms of $\mathrm{k(C)}$ and the Laplacian eigenvalues $\lambda_1 = 0, \lambda_2, \dots, \lambda_N$:

$$\mu_i = \mu_i(\mathrm{C}) = \frac{\sigma^2}{2(\mathrm{k(C)} - \lambda_i)}(\mathrm{e}^{2(\mathrm{k(C)}-\lambda_i)\Delta \mathrm{t}} - 1)\mathrm{e}^{2(\mathrm{k(C)}-\lambda_i)t} \qquad \text{(S-4)}$$

While we conjecture that the same invariance hold assuming non-identical returning forces, we are only able to derive a closed-form proof for the special case of homogeneous returning forces.

## 2. Invariance of leading covariance eigenvalue and structure revealing property at transition verified on large-scale random networks

Many real-world complex networks have internal feedbacks that can potentially facilitate or inhibit the network's evolution toward a transition. Figure S1 verifies the property 2—the invariant effect of global feedbacks. Three different networks (linear, 5-4 clustered, and fully connected) go through pitchfork bifurcation driven by a feedback of an 'order parameter' given by: $\frac{\mathrm{dC}}{\mathrm{dt}} = \frac{\mathrm{X} \cdot v_{sync}}{\|\mathrm{X}\|}$. The RHS term measures the state change projected to the synchronization direction $v_{sync}$, normalized by the magnitude of the state. Each network's evolution to a bifurcation is triggered by a momentary synchronization of random state changes. However, once initiated, the evolution advances steadily regardless of the network connectivity. The increase of the control parameter C follows the same linear slope (the top plots) and the state changes go through the same curve (the bottom plots).

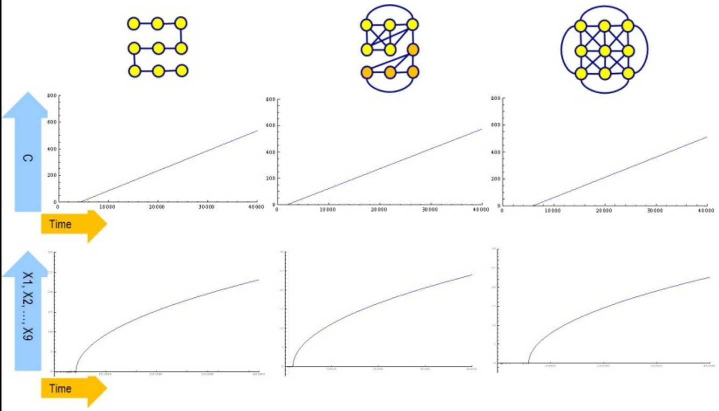


Figure S1: **Network invariance of synchronization-induced bifurcation. (Top row)** The evolution of the phase parameter C. The horizontal and vertical axes represent the evolution of the networks toward a bifurcation and the phase parameter C, respectively. **(Bottom row)** The horizontal and vertical axes represent the evolution of the networks toward a bifurcation and the state changes of X, respectively. The bifurcation is triggered by random synchronization, but once kicked, in follows the same schedule regardless of the network connectivity.

## 3. Invariance of leading covariance eigenvalue and structure revealing property at transition verified on large-scale random networks

Tests on large-scale random networks also reveal the same behavior near the bifurcation. Both a Watts-Strogatz small-world network and a Barabasi-Albert scale-free network with 400 nodes have been tested and the results are summarized in Figure S2. The covariance spectrums show the expected invariance property of $\mu_1$ and variance property of the rest of the spectrum. The

lower spectrums reveal stark difference between the small-world network (top: spectral gap = 0.073) and the scale-free network (bottom: spectral gap = 0.535). The small-world network reveals its structure near the bifurcation. The locally connected neighbors appear as horizontal strips (the network nodes have 'raster scan' type order in the pixel domain) in the pixel representation, and occasional thick strips capture the long range correlation due to the non-local links that equip the network the small-world property. On the other hand, the scale-free network, being relatively dense and free of scale structure, does not reveal much structure.

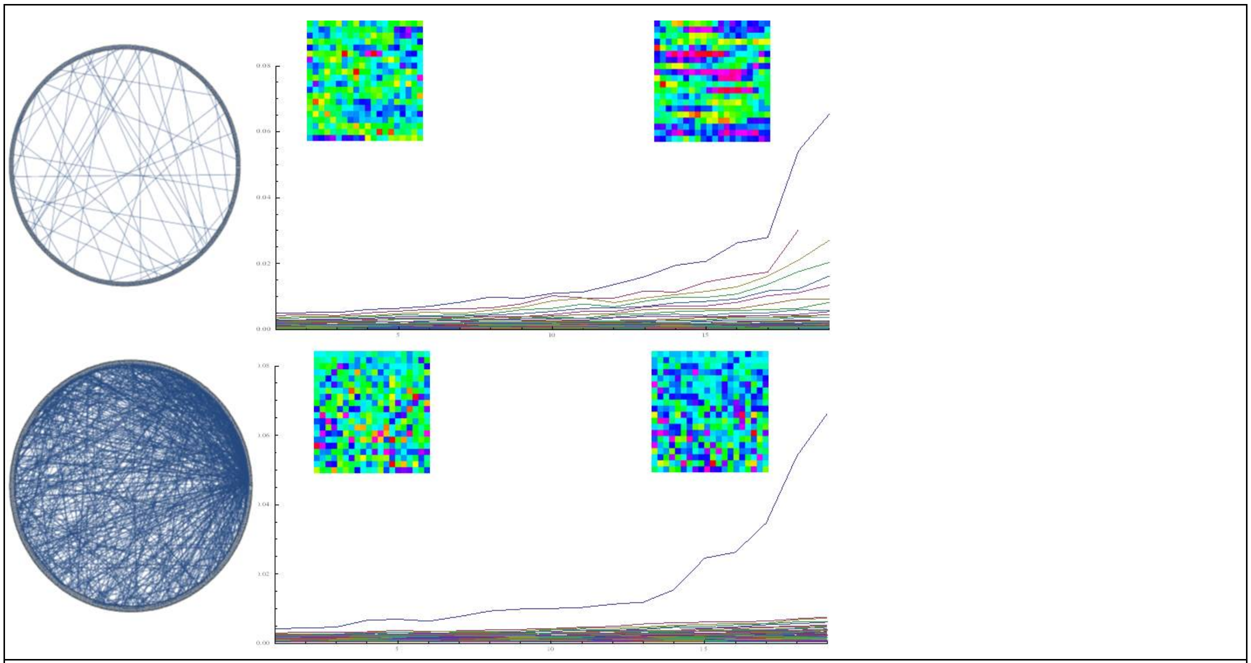

Figure S2: **Invariance ($\mu_1$) and pattern revealing properties for a small-world network (top) and a scale-free network (bottom).** The horizontal and vertical axes represent the evolution of the networks toward a bifurcation and the eigenvalues, respectively. The inset pictures compare 20x20 node states as patterns in 20x20 pixel values. The locally connected neighbors of the small-world network appear as horizontal strips in the pixel representation, and occasional thick strips capture the long range correlation due to the non-local links that equip the network the small-world property. The structure is amplified near the phase transition. The scale-free network does not reveal much structure either far from the transition, or near the transition.

## 4. Market forecasting methodology

We provide details on how we utilize $\mu_1$ for making stock market forecasts. We calculate the short-term market correlations and the long-term correlations, then combine them as a measure of overall market instability. Given the 12-dimensional time series of Dow Jones component share prices $Z(t) = (z_1(t), z_2(t), \ldots, z_{12}(t))$, we compute their short-term and long-term returns as state variables with 1-day return window (daily return) for capturing short-term dynamics and 70-day return window for capturing long-term dynamics:

$x_i^{short}(t) = x_i^s(t) = z_i(t)/(z_i(t-1)$ and $x_i^{long}(t) = x_i^l(t) = z_i(t)/z_i(t-70)$

Here, $i = 1,..,12$ and let's define $X^s(t) = \{x_i^s(t)\}_{i=1,..,12}$, $X^l(t) = \{x_i^l(t)\}_{i=1,..,12}$. We then compute the first leading covariance eigenvalue $\mu_1(t) = \mu_1^s$ (t) capturing the short-term market correlations from the covariance matrix $Cov_{t,t+10}(X^s)$ over the moving time window $[t, t+10]$, and the second covariance eigenvalue $\mu_1(t) = \mu_1^l(t)$ capturing the long-term correlations from the covariance matrix $Cov_t(X^l)$. Note that we do not apply time window for the long-term dynamics, because the long-term effect has been already accounted for by the 70-day change. In effect, this is a matrix capturing pairwise correlation of long-term changes: $Cov_t(X^l)_{i,j} = E(\frac{z_i(t)}{z_i(t-70)}\frac{z_j(t)}{z_j(t-70)})$. Then the final indicator is: $\mu_1(t) = \mu_1^s(t) + \mu_1^l(t)$.

The daily calculated $\mu_1(t)$ is compared to a threshold to make a prediction based on a simple decision function for a D-day ahead prediction: generate warnings of 'P% crash D-days from now' if $\mu_1(t) > threshold$. For generating performance curves (ROC) as shown in Figure 6, we regard the prediction as a detection problem so that TPR = (# of correctly predicted events)/(# of P% true crash events) and FPR = (# of falsely predicted events)/(# of generated predictions).

**5. Persistent pattern dynamics before and during major stock market crises**

We provide examples of persistent pattern dynamics before and during major stock market crashes in addition to the 2008 crash example in the main text. The 1987 'Black Monday' event and the most recent major crash happened in 8/11/2011 show similar pattern dynamics (Figures S3 and S5). However, the stock market crash due to the 9/11 terrorist attack, Figure S4) does not show the signature behavior of persistent large-scale correlation of share prices observed during 1987, 2008, and 2011 crashes.

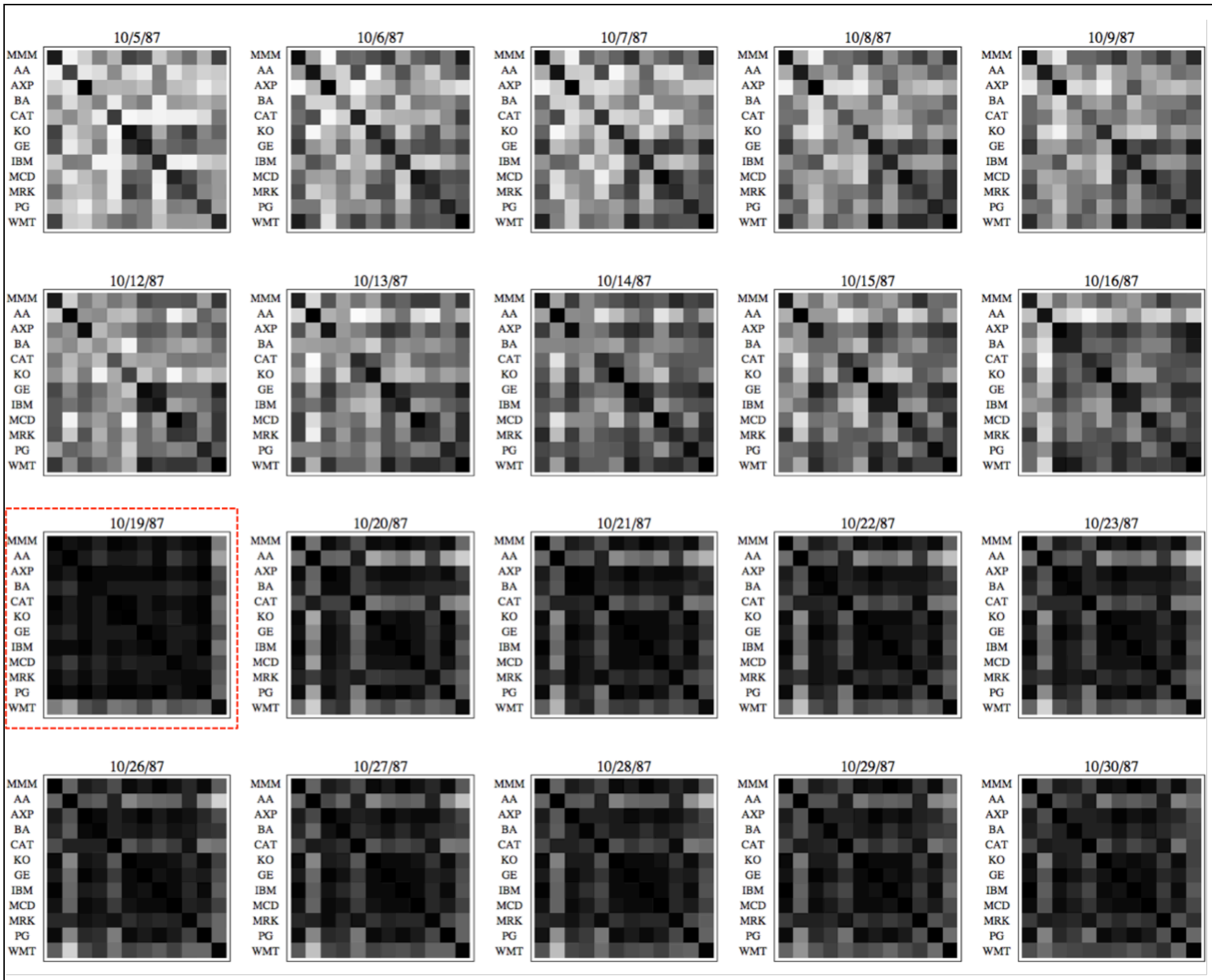

Figure S3: **Correlation pattern dynamics as indicators during the 1987 'Black Monday' crash.** The crash (DJIA losing 22.7% in a single day of 10/19/1987) is preceded by gradual build-up of market correlations, clearly observed in 10/16, the prior Friday. The dotted red square marks the day of the crash.

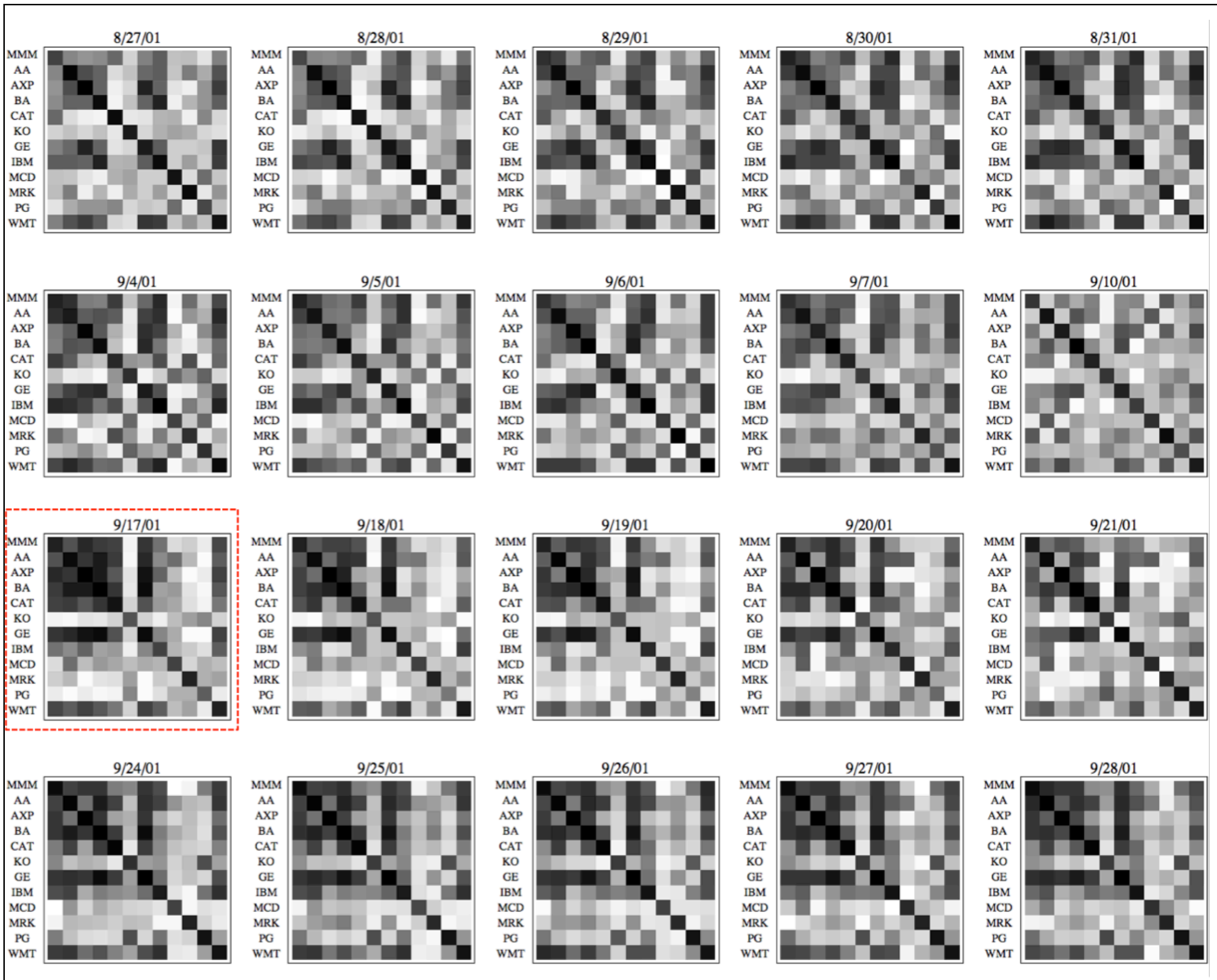


Figure S4: **Persistent correlation pattern dynamics absent during the 9/11 market crash** The market crash (the market closed 9/11 and reopened 9/17) was triggered by an external event, and the market dynamics does not show the pattern of persistent large-scale correlation. The Dow Jones index quickly recovered to the level before the attack after a few weeks, unlike other major 'endogenous' market troubles.

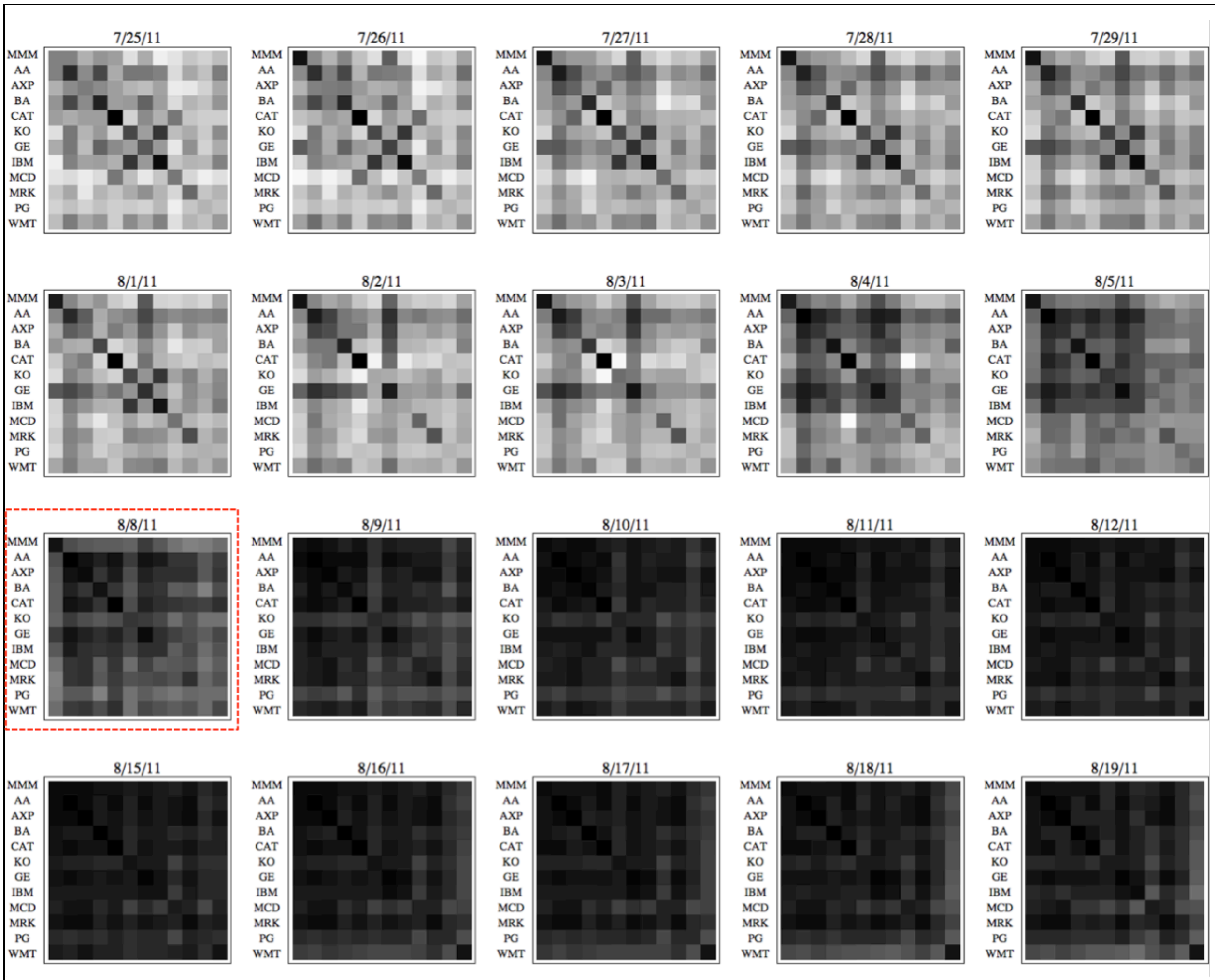


Figure S5: **Correlation pattern dynamics as indicators of large-scale market crash** The most recent large-scale US stock market crash happened 8/11/2011, mainly triggered by the Standard and Poor's downgrade of US credit rating.
